# Late spontaneous posterior capsule rupture with single-piece hydrophobic acrylic intraocular lens dislocation

**DOI:** 10.1038/s41598-024-53934-z

**Published:** 2024-02-09

**Authors:** Soomin Lee, Gahye Lee, Choul Yong Park

**Affiliations:** Department of Ophthalmology, Dongguk University, Ilsan Hospital, 814, Siksadong, Ilsan-dong-gu, Goyang, 410-773 Gyunggido South Korea

**Keywords:** Dislocation, Displacement, Intraocular lens, Posterior capsule, Rupture, Spontaneous, Lens diseases, Refractive errors

## Abstract

In this study, we described and discussed the late onset spontaneous posterior capsule rupture with intraocular lens (IOL) dislocation years after uncomplicated cataract surgery and implantation of hydrophobic acrylic IOLs. Eight patients presented with spontaneous posterior capsule rupture and IOL dislocation 5–20 years after uncomplicated phacoemulsification and IOL (AcrySof, Alcon, US) implantation. None of the patients had undergone posterior capsulotomy in the past. Four of the patients admitted habitual eye rubbing. An intact and well-centered continuous curvilinear capsulotomy edge was observed in all cases. IOLs were dislocated or displaced behind the anterior capsulotomy with a significant decrease in vision. A large rupture with a curled edge of the broken posterior capsule was visible. Dislocated IOLs were removed, and a three-piece IOL was inserted in the sulcus in six cases and suture fixated to the sclera in two cases. Improved vision was achieved in all cases. Although the mechanism underlying this late complication is unclear, habitual eye rubbing or IOL design may play a role. Further investigation is needed to prevent this complication in the future.

## Introduction

Intraocular lens (IOL) dislocation or displacement after uncomplicated cataract surgery reportedly occurs in about 1–2% of cases^1–3^. A dislocated IOL results in a significant decrease in vision and various ocular complications, such as inflammation, glaucoma, retinal detachment and macular edema^4–6^. Most cases of late IOL dislocation are “in the bag dislocation”^7^. IOL rectification or exchange in the absence of posterior capsule support is a challenging procedure for many cataract surgeons.

The late spontaneous rupture of the posterior capsule with IOL dislocation is a relatively rare complication, and only fewer than 20 cases have been reported^8–10^. Although this unique complication has recently received attention and some cases have been reported, it may be that we have paid less attention to it because it is hidden in the shadow of the broader diagnosis of IOL dislocation. In the past 20 years, foldable IOLs have been commonly used in routine cataract surgery, and a sharp square edge of the optic and haptic of the IOL has been favored to prevent the occurrence of late posterior capsular opacification^11^. However, there may be hidden risk factors for the spontaneous rupture of the posterior capsule that we have not noticed in the process of IOL development.

In this study, eight cases of late spontaneous rupture of the posterior capsule with hydrophobic IOL dislocation and the possible underlying mechanism are discussed. Because foldable hydrophobic posterior chamber IOLs (AcrySof Alcon, Fort Worth, US) have been mainly used in our institution for many years, the presented cases in this study all used the hydrophobic IOL from the same brand.

## Results

The median age of eight patients was 56 years (range 33–64 years) and they were all males. The median implantation duration of the dislocated IOLs was 10 years (range 5–20 years). The median age at which the dislocated IOLs were implanted was 46 years (range 23–54 years). Axial length of the diseased eye was measured as 25.30 mm (range 22.78–26.68 mm).

Tables 1 and 2 show the clinical characteristics of the study eyes and the comparison of biometric parameters between the study eyes and the contralateral control eyes.

**Table 1:** Clinical characteristics of the presented cases. Habitual eye rubbing refers to patients answering ‘yes’ when asked whether they have a habit of rubbing eyes frequently.

Case No	Age/Sex	Laterality	Race	Length of implantation (years)	Intraocular lens	Ocular comorbidity	Habitual eye rubbing	Preoperative fragmented and curled posterior capsule debris
1	54/M	Right	Asian	9	AcrySof SN60AT	None	Yes	Yes
2	33/M	Left	Asian	10	AcrySof IQ SN60WF	None	Yes	Yes
3	40/M	Right	Asian	6	AcrySof IQ SN60WF	None	Yes	Yes
4	60/M	Left	Asian	12	AcrySof IQ SN60WF	None	No	Yes
5	64/M	Right	Asian	20	AcrySof SN60AT	Chronic open angle glaucoma	No	Yes
6	58/M	Left	Asian	10	AcrySof IQ SN60WF	Diabetic retinopathy, Chronic open angle glaucoma, panretinal photocoagulation, trabeculectomy	Yes	Not available due to very small and rigid pupil
7	59/M	Right	Asian	5	AcrySof ReSTOR SN6AD1	None	No	Yes
8	49/M	Right	Asian	11	AcrySof ReSTOR SN6AD1	Previous LASIK	No	Yes

**Table 2:** The comparison of biometry between study eyes and contra-lateral eyes. K1: flat keratometry, K2: steep keratometry, Km: mean keratometry, ACD: anterior chamber depth, AXL: axial length, D: diopter, NA: not available.

Case No	Study eye					Contra-lateral eye				
	K1 (D)	K2 (D)	Km (D)	ACD (mm)	AXL (mm	K1 (D)	K2 (D)	Km (D)	ACD (mm)	AXL (mm)
1	44.94	46.30	45.62	NA	22.78	45.06	45.92	45.49	3.43	23.89
2	44.41	45.79	45.10	NA	25.45	43.95	45.06	45.51	4.00	25.78
3	42.83	43.38	43.11	NA	25.64	42.94	43.66	43.30	4.87	24.99
4	40.61	41.25	40.93	7.00	25.36	40.86	41.19	41.03	3.24	25.38
5	41.71	43.20	42.46	NA	24.03	41.95	42.95	42.45	2.63	24.06
6	23.20	42.53	43.81	4.46	23.20	NA	NA	NA	NA	NA
7	43.05	43.98	43.52	5.57	25.25	42.95	43.67	43.31	4.73	25.25
8	42.10	42.66	42.38	NA	25.82	40.61	41.10	40.86	4.01	25.78

The originally implanted IOLs are single piece hydrophobic acrylic IOL with sharp square edged haptic design. Two eyes were implanted with spherical IOL (AcrySof SN60AT), four eyes with aspherical IOL (AcrySof IQ SN60WF) and the other two eyes were implanted with multifocal aspherical IOL (AcrySof ReSTOR SN6AD1). One patient (case 6) had the history of bilateral LASIK surgery 16 years before the cataract surgery. Two patients (case 5 and case 6) were diagnosed as chronic open angle glaucoma and under the daily topical anti-glaucoma medication.

All patients are male, and four of them admitted habitual eye rubbing. Interestingly, seven out of eight cases (except case 7) had cataract surgery on only one eye and were presenile cataracts in their 30 s or 40 s. Neodymium-Yttrium–Aluminum-Garnet (Nd-YAG) posterior capsulotomy had not been performed in any of the cases.

**Figure 1:**
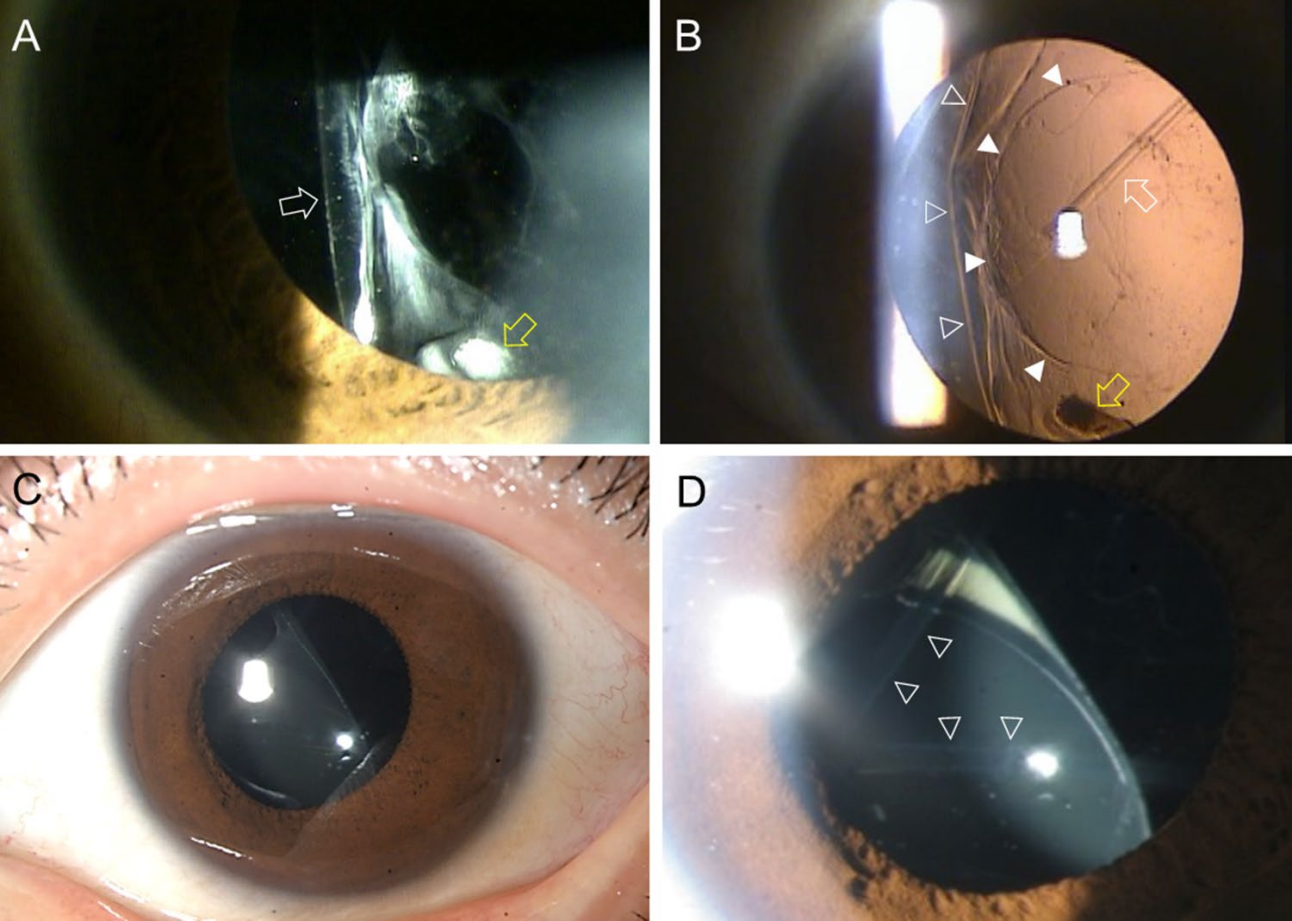
Slit lamp photographs of case 1 (**A**,**B**) and case 2 (**C**,**D**). (**A**) The displaced lens capsule margin (arrow) and one haptic tip (yellow arrow) are visible with zonulysis. (**B**) One haptic tip is clearly visible at the inferior pupil margin (yellow arrow) with the IOL dislocated into the anterior vitreous cavity. The debris of the broken posterior capsule is curled (white arrow). The displaced lens capsule margin (blank arrowheads) and continuous curvilinear capsulotomy edge (white arrowheads) are clearly visible. (**C**) The IOL is displaced in the inferior nasal direction. (**D**) The curled edge of a large V-shaped posterior capsule rupture is clearly visible (arrowheads).

**Figure 2:**
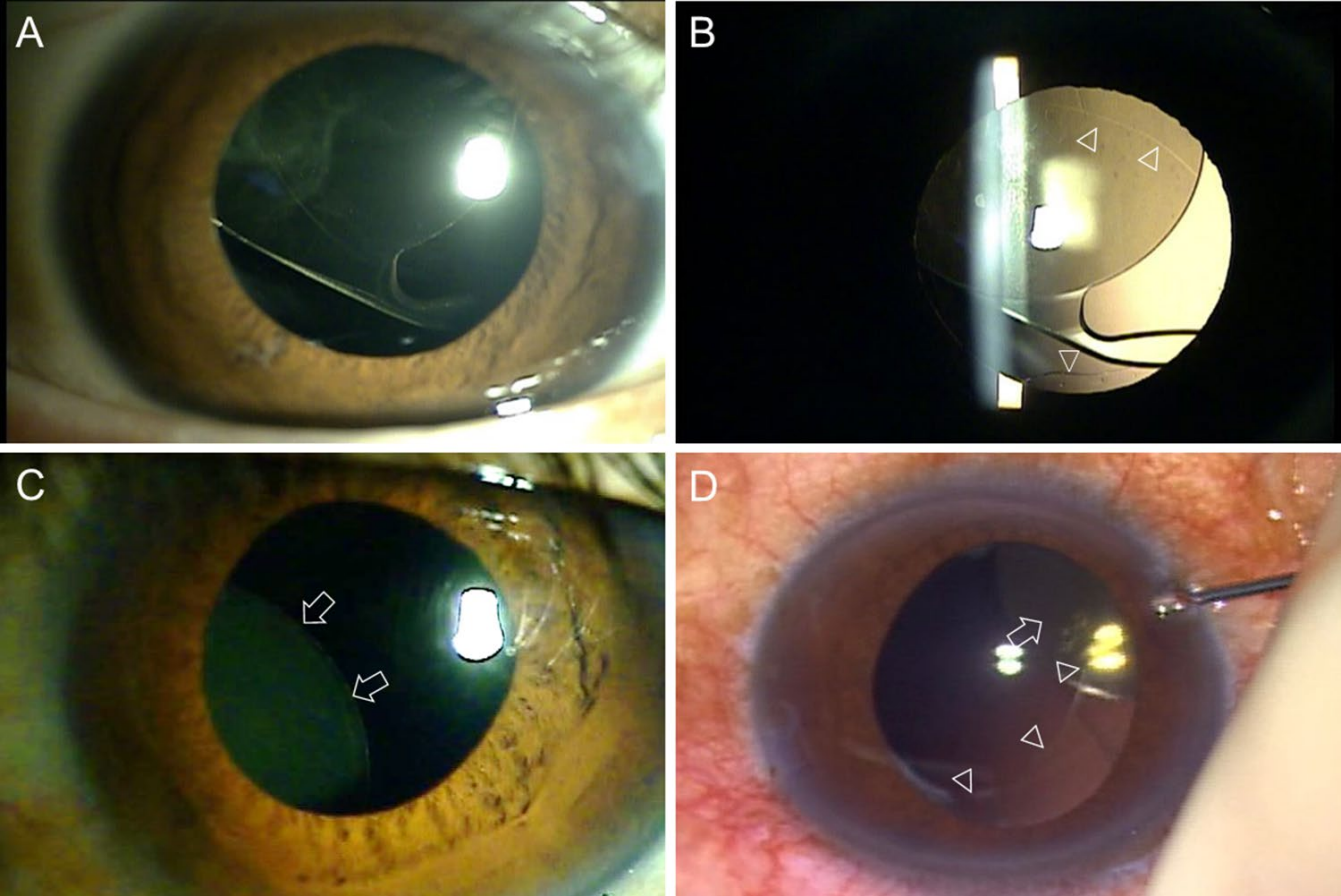
Slit lamp photographs of case 3 (**A**,**B**) and case 4 (**C**,**D**). (**A**) The superiorly displaced IOL is visible. (**B**) Intact well-centered continuous curvilinear capsulotomy is visible (arrowheads). (**C**) An inferior nasally displaced IOL optic is visible (arrows). (**D**) The operation view reveals an intact continuous curvilinear capsulotomy edge (arrowheads) with a displaced IOL (arrow).

**Figure 3:**
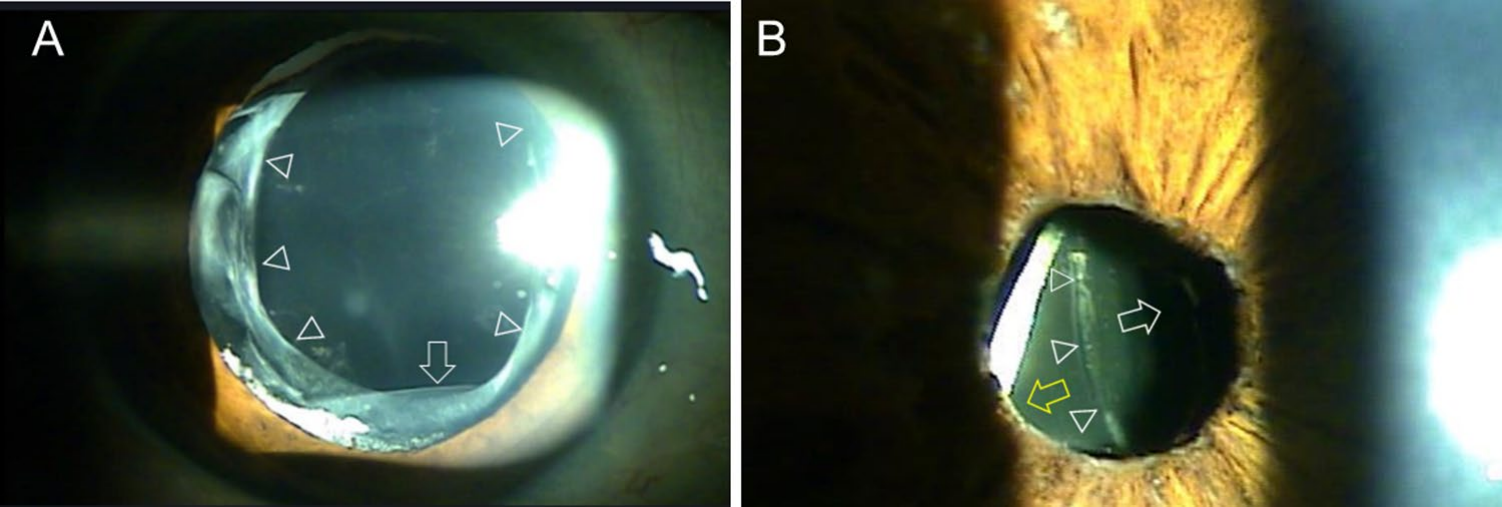
Slit lamp photographs of case 5 (**A**) and case 6 (**B**). (**A**) The impression of the haptic with the Soemmering’s ring is visible at 10 o’clock position. The haptic of inferiorly dislocated IOL is visible (arrow) with intact continuous curvilinear capsulotomy edge (arrowheads). (**B**) Temporal displacement of IOL is visible. The groove (arrowheads) dividing optic (white arrow) and haptic (yellow arrow) is bisecting the pupil.

**Figure 4:**
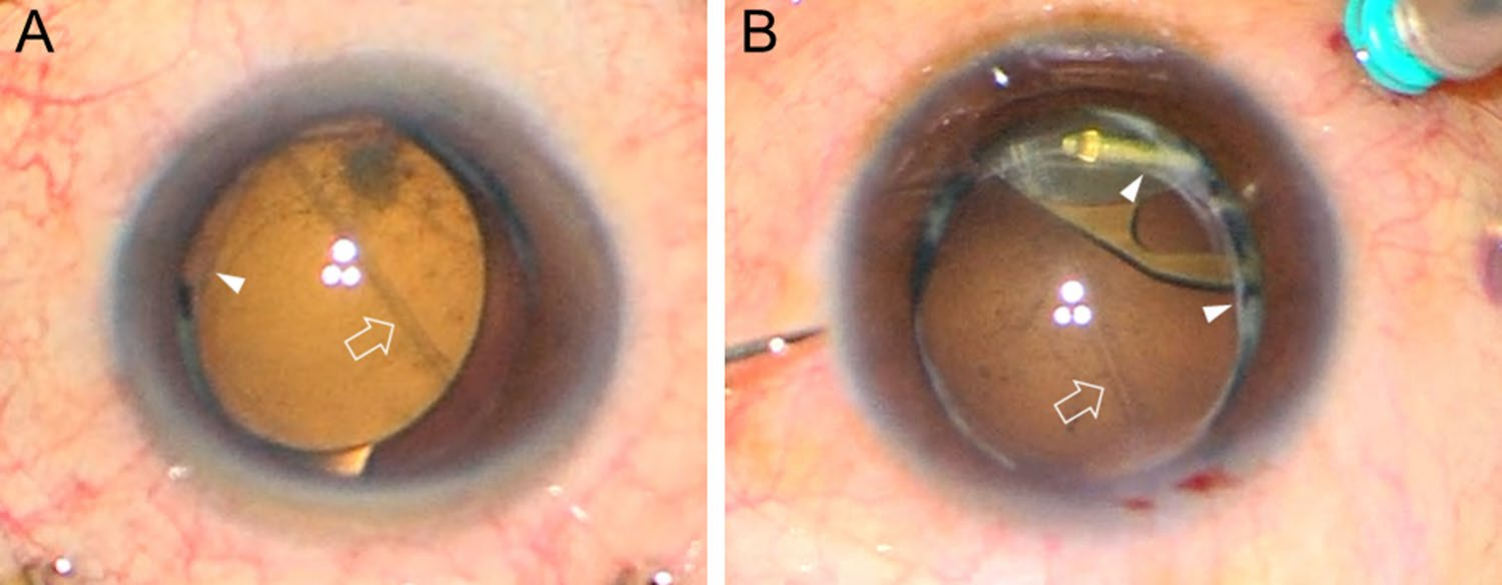
Image capture from the surgical videos of case 7 (**A**) and case 8 (**B**). Intact well-centered continuous curvilinear capsulotomy is visible (arrowheads). The debris of the broken posterior capsule is curled and floating in the anterior vitreous. (white arrow).

The anterior segment photos of all cases were presented in the figures. On slit lamp examination, it was confirmed that IOL was separated from the capsule remnant and dislocated into the anterior vitreous. The presence of broken posterior capsule margin or fragmented/curled posterior capsule debris in the anterior vitreous cavity are the characteristic finding of this disease entity and was observed in 7 out of 8 cases (Fig. 1). These posterior capsule debris often appeared very strongly rolled up. Intact well-centered continuous curvilinear capsulotomy was visible in six cases (Figs. 2, 3A, and 4). Significant zonular defect was observed by the slip lamp in case 1 and at the operating table in case 6. (Figs. 1A,B and 3B). In all cases, visual improvement was achieved by removing the dislocated IOL and inserting a three-piece IOL in the sulcus (cases 2, 3, 4, 5, 7 and 8) or by scleral fixation (case 1 and 6).

After IOL exchange, patients were followed up for at least 12 months–6 years, and it was confirmed that secondary implanted IOLs remained stable during this period.

## Discussion

As demonstrated in this study, IOL displacement or dislocation was accompanied by a large tear in the posterior capsule, which remained intact for years after the cataract surgery. As well known, the late localized break of the posterior capsule generally has little effect on IOL centration, as has been shown in many cases of Nd-YAG capsulotomy after cataract surgery. The adhesion between the anterior and posterior leaflets around the haptic of the IOL that was formed over months to years, usually supports the IOL optic and prevents IOL displacement. Therefore, the IOL displacement in our cases may have different underlying mechanisms. Because habitual eye rubbing was reported in four of our cases, a repeated and strong external force applied to the IOL may have played an important role in the spontaneous rupture of the posterior capsule.

Excessive eye rubbing has been suspected to be a risk factor for late posterior capsule ruptures in previous reported cases. Chen et al.^9^ reported a case of bilateral posterior capsule rupture in a 71-year-old male 11 years after uncomplicated cataract surgery with bilateral Alcon AcrySof SN60WF IOL implantation. The curled capsular flap was detected in the area of the ruptured posterior capsule. The authors suspected vigorous habitual eye rubbing to be the cause of the late spontaneous rupture of the posterior capsule. Bassily et al.^10^ reported a case of a 36-year-old woman with a bilateral late spontaneous posterior capsule rupture and IOL dislocation 9 years after uncomplicated cataract surgery with bilateral implantation of three- piece silicone IOLs (Ceeon 911A, Abbott). The curled edge of the posterior capsule was also observed in this case. The patient suffered from severe eczema and engaged in vigorous eye rubbing. The authors suspected the habitual eye rubbing induced the posterior capsule rupture because the focal digital pressure in the eye might displace the IOL vertically, causing the sharp edge of the haptic to rupture the posterior capsule. Eye rubbing of a pseudophakic eye can also induce “in-the–bag IOL dislocation”. Yamazaki et al.^12^ reported the partial rupture of zonule and anterior displacement of IOL /lens capsule complex in a 66- year- old man with atopic dermatitis and habitual eye rubbing. Peter et al.^13^ reported a case of repeated anterior subluxation of IOL/lens capsule accompanied by a sectoral zonular defect after habitual eye rubbing.

More recently, Yehezkeli et al.^8^ reported four cases of late spontaneous posterior capsule rupture and IOL dislocation after implantation of a hydrophilic single- piece IOL. All four cases were symptom-free for 17–20 years after uncomplicated cataract surgery before the onset of IOL complications. The patients denied any ocular trauma. The habitual eye rubbing was not investigated. All the IOLs were dislocated through a large posterior capsule break (equator to equator). Of note, the torn posterior capsules in these cases were transparent, with no evidence of fibrosis. The authors proposed that an excessively bent haptic of a certain foldable single-piece IOL might at some point exceed the posterior capsule resistance and lead to a rupture and IOL dislocation^8^.

Culp et al.^14^ reported the largest case series (eight cases) of late spontaneous posterior capsule rupture and IOL dislocation. Although their report presented 10 cases of IOL dislocation, late spontaneous posterior capsule rupture was uncertain in two cases^14^. After the pathologic analysis of explanted IOLs and capsules, they proposed “dead bag syndrome” as a condition with characteristics, such as absent lens epithelial cells and fibrosis, and underlying capsule splitting or thinning. A patient with dead bag syndrome may experience spontaneous posterior capsule rupture even after minor trauma due to the increased fragility of the posterior capsule.

This complication causes serious visual loss, and our cases and previous cases show that it is not as rare as expected. However, the exact underlying mechanism has not yet been established, and risk factors can be inferred through case studies.

The design of IOLs can be another risk factor for the late posterior capsule rupture. The previously reported cases of spontaneous rupture of posterior capsules and IOL dislocation are summarized in Table 3. Regardless of IOL material, structure (single-piece or three-piece), and implantation duration, spontaneous posterior capsule rupture with IOL dislocation occurred. However, it is of note that there has been no case report of spontaneous posterior capsule rupture in IOLs with a plate haptic design, unlike with a C loop or a J loop design.

**Table 3:** Previous reports of late posterior capsule rupture and IOL dislocation.

Author	Age/sex		Laterality	Intraocular lens (maker)	IOL material	Length of implantation (years)	Comorbidity
Bassily et al	36/F		Bilateral	Ceeon 911 (Abbott Medical Optics, Inc.)	Silicone, three-piece	9	Atopy and eczema
Chen et al	71/M		Bilateral	AcrySof SN60WF (Alcon)	Hydrophobic acryl, single- piece	11	Allergic conjunctivitis
Yehezkeli	4 cases:	70/M 75/M 54/M 68/M	Unilateral	B-Lens( Hanita Lenses)	Hydrophilic acryl, single- piece	17–20	Not available
Culp et al	8 cases:	72/M, 66/M, 68/M	Unilateral	SI30 (Abbott )	Silicone, three-piece	13–18	Not available
		62/M, 56/M	Unilateral	Tecnis (Abbott)	Hydrophobic acryl, single- piece	3.7–4	Not available
		60/F	Unilateral	AcrySof SN60WF (Alcon)	Hydrophobic acryl, single- piece	Not available	Not available
		57/M	Unilateral	CQ2015A (STARR Surgical)	Collagen copolymer, three-piece	Not available	Not available
		75/M	Unilateral	AcrySof SN60AT (Alcon)	Hydrophobic acryl, single- piece	12.3	Not available

Considering the findings of our cases and those of others, the underlying mechanism of late posterior capsule rupture may be different from that of the “in-the-bag IOL dislocation”. The “in-the-bag IOL dislocation” has been far more commonly reported than late posterior capsule rupture with IOL dislocation^7,15,16^. Pseudoexfoliation syndrome has been suspected as the most common reason for late “in-the-bag IOL dislocation”^7,17^. Surgical trauma to the zonule, axial myopia, and uveitis are also known to be risk factors for late “in-the-bag IOL dislocation”^7,18^. These risk factors increase zonular weakness. However, the risk factors for “in-the-bag IOL dislocation” were not found in our cases. Although it is not consistent in all cases, habitual eye rubbing, dead bag appearance, sticky IOL material, design and a sharp square haptic edge may have a combination effect on the development of late spontaneous posterior capsule rupture. The possible hypothesis is that the sticky nature of modern IOL material makes the sharp square haptic edge firmly attach to the weak point of posterior capsule which has the feature of “dead bag syndrome” and a habitual eye rubbing generates small but repeated pressure and induces an initial tiny break of the posterior capsule. The small break continues expand with further eye rubbing until IOL instability develops. In addition, the excess movement of unstable IOL may induce further damage to zonules observed in two of our cases.

Our study found that five out of eight eyes had axial length over 25.00 mm, which is in the myopic range. High myopia and ocular trauma has been reported as risk factors for the IOL dislocation following routine cataract surgery^19^. However, in the eyes of this study, the axial length did not fall in the range of high myopia and was not significantly longer than that of the contra-lateral eye. Case 6, unlike the other eyes, had a history of trabeculectomy and panretinal photocoagulation using contact lens. Trauma from these procedures may also increase the risk of IOL instability.

It is interesting that almost all cases, including ours and others in Table 3, had no history of Nd-YAG capsulotomy. If late posterior capsule rupture is the result of localized pressure caused by a sharp square edge of the haptic or optic, the existence of posterior capsulotomy may serve as a ventilation route to relieve some of the externally applied pressure and the fluid flow through the posterior capsulotomy may act as a buffer between the IOL and the posterior capsule. Another possibility is that reduction in the posterior capsule tension by Nd-YAG capsulotomy may be effective in preventing late posterior capsule rupture. However, it is difficult to prove this hypothesis unless the incidence of IOL dislocation similar to this study is compared in eyes that did not receive Nd-YAG capsulotomy and in eyes that received Nd-YAG capsulotomy. Additionally, when Nd-YAG capsulotomy is performed, it may be more difficult to detect even if a larger posterior capsule rupture occurs later due to the IOL.These possibilities can be elucidated through more case observations and experiments in the future.

It is noteworthy that of the 22 cases reported so far (14 cases from previous reports and 8 cases from this study), 20 cases were male and 2 cases were female. This means that the disease may occur more frequently in males, but the exact mechanism needs further investigation. The age at which cataract surgery was performed is also interesting. Seven out of our 8 cases underwent initial cataract surgery at or under age of 50, and the remaining one case also underwent surgery at the age of 54, which are relatively younger than the typical cataract surgery age. The effect of age and gender on this characteristic IOL dislocation needs further study.

The limitation of this study is that it was not possible to find the exact underlying pathology through a small number of cases. Although various possibilities have been suggested, it is necessary to identify the exact cause through additional large-scale investigation. It is known that surgical details, including type of the cataract, size of the capsulotomy, surgical technique of nucleus removal, type of the viscoelastic material and posterior capsule polishing may affect the IOL long-term stability after the routine cataract surgery. Unfortunately, most of our cases underwent the primary IOL implantation at local primary ophthalmology clinics. Therefore, detailed information about the surgery was not available. Another limitation of this study is that we could not reveal whether the details of the surgical procedure increased the risk of IOL dislocation of the current cases. Additionally, this study only analyzed cases of complications caused by a single type of IOL design with the same geometry and material. This analysis method has the advantage of being able to exclude the influence of various IOL materials or designs on IOL dislocation, but it also has the disadvantage of having a relatively small number of cases.

This study is also unique in the it is the largest case series to date collecting only late spontaneous rupture of posterior capsule and IOL dislocation caused by the same hydrophobic IOLs manufactured by a single company.

In summary, we are now aware that spontaneous posterior capsule rupture is a rare complication, but it does occur. Nevertheless, there is still insufficient evidence to clearly understand the mechanism underlying this serious and late complication. Therefore, more attention and further investigation are needed to elucidate the exact pathogenesis of this complication and to develop effective prevention methods.

## Methods

This study followed the tenets of the Declaration of Helsinki and was approved by the institutional review board of Dongguk University, Ilsan Hospital, Goyang, South Korea (IRB No. 2002–05-038). Informed consent was received to publish the case description and any accompanying images from the patients. The medical records of patients who underwent IOL rectification or exchange surgery at Dongguk University, Ilsan Hospital between January 2015 and April 2023 were reviewed. Out of 124 cases of total IOL rectification or IOL exchange cases, eight cases were confirmed to have the late spontaneous rupture of posterior capsule and IOL dislocation years after initial uncomplicated cataract surgery confirmed through medical record, anterior segment photos and surgical video reviews.

The confirmation criteria of the late spontaneous rupture of posterior capsule and IOL dislocation was as follows. The diagnosis was made when all the criteria were met.

1. Confirmed medical record of initial cataract surgery with successful in the bag IOL implantation.
2. IOL dislocation was diagnosed at least 1 year after the first cataract surgery.
3. No history of ocular trauma or surgery after the first cataract surgery.
4. The presence of posterior capsule fragment or curled posterior capsule debris in the anterior vitreous cavity was confirmed on the slit lamp examination.
5. IOL was dislocated from the anatomical plane.
6. Intact anterior continuous curvilinear capsulotomy was visible at the anatomical plane on the slit lamp examination

### Ethical approval

This study followed the tenets of the Declaration of Helsinki and was approved by the institutional review board of Dongguk University, Ilsan Hospital, Goyang, South Korea (IRB No. 2002-05-038).

### Consent to participate

Informed consent was obtained from all individual participants included in this study.

## Data Availability

All the data of the current study are available from the corresponding author on reasonable request.
